# Remote Conditioning by Rhythmic Compression of Limbs Ameliorated Myocardial Infarction by Downregulation of Inflammation *via* A2 Adenosine Receptors

**DOI:** 10.3389/fcvm.2021.723332

**Published:** 2022-04-08

**Authors:** Senlei Xu, Renjun Gu, Xiangyu Bian, Xin Xu, Xuefeng Xia, Yuchen Liu, Chengjie Jia, Yihuang Gu, Hongru Zhang

**Affiliations:** ^1^School of Acupuncture and Tuina, School of Regimen and Rehabilitation, Nanjing University of Chinese Medicine, Nanjing, China; ^2^Key Laboratory of Acupuncture and Medicine Research of Ministry of Education, Nanjing University of Chinese Medicine, Nanjing, China; ^3^The First School of Clinical Medicine, Nanjing University of Chinese Medicine, Nanjing, China; ^4^Wuxi Municipal Rehabilitation Hospital, Wuxi, China; ^5^School of Chinese Medicine, School of Integrated Chinese and Western Medicine, Nanjing University of Chinese Medicine, Nanjing, China

**Keywords:** remote tissue compression, myocardial infarction, adenosine, A2 receptor, signal transduction, anti-inflammatory

## Abstract

**Background:**

Remote ischemic conditioning (RIC) is a cardioprotective phenomenon, yet transient ischemia is not a requisite trigger for remote cardioprotection. In fact, RIC is a stimulus compound containing interruption of the blood vessel and tissue compression. In this study, we evaluate the effects of remote tissue compression on infarct size after myocardial infarction and explore its preliminary mechanisms.

**Methods and Results:**

We used a murine model of myocardial infarction to assess ischemia injury and identified remote conditioning by rhythmic compression on forelimb as a novel cardioprotective intervention. We show that the cardioprotective signal transduction of remote conditioning from the trigger limb to the heart involves the release of adenosine. Our results demonstrate that A2a and A2b receptors are indispensable parts for cardioprotection of remote conditioning, which is linked to its anti-inflammatory properties by the subsequent activation of cAMP/PKA/NF-κB axis.

**Conclusion:**

Our results establish a new connection between remote tissue compression and cardiovascular diseases, which enhances our cognition about the role of tissue compression on RIC cardioprotection.

## Introduction

Acute myocardial infarction (AMI) is a condition of acute ischemic necrosis of myocardial tissues, which contributes significantly to morbidity and mortality on a global scale (1). Over the past 3 decades, various strategies have been developed to protect the heart against AMI (2). Remote ischemic conditioning (RIC), especially, has been established in many experimental studies and shown to be cardioprotective. Importantly, RIC by brief episodes of ischemic or reperfusion on an extremity reduces infarct size and improves the prognosis of AMI patients (3). Regarding RIC, a variety of evidence from a spectrum of models suggests that the interruption of blood flow is not an essential trigger for remote cardioprotection (3). Apart from the cycles of transit ischemia, RIC also involves mechanical stimuli through tissue compression (3). However, the role of tissue compression in cardioprotection of RIC is rarely studied.

Indeed, mechanical manipulation of body tissues by rhythmical compression involves the immune system and that this can be used as an adjunct treatment for the variety of mental and physical conditions (4, 5). The recent experimental evidence suggested that cycles of compression temper the increase in the number of cells expressing pro-inflammatory cytokines, tumor necrosis factor-α (TNF-α), and monocyte chemoattractant protein-1(MCP-1) (6). It has been demonstrated that tissue compression acts on mechanoreceptors in the skin to influence the release of soluble messengers that are considered responsible for mediating the immune response (7). Specifically, chiropractic manipulation and massage might be associated with an efflux of cytosolic ATP that is sufficient to elevate extracellular adenosine, which is also a critical trigger and also a mediator in RIC-induced cardioprotection (8–10). Adenosine is a master regulator of energy metabolism in an emerging paradigm of immunity, where recognition of cell stress initiates and inhibits inflammation (11). In the previous studies, we found that the reduction of myocardial inflammation was associated with decreased infract size and improved cardiac function (12). Of the 4 adenosine receptor subtypes, A2 receptors are proposed to act as the triggers of the emergency downregulation of overactive inflammatory response (13). What is more, some studies directly showed that adenosine analogs selectively targeting A2 receptors are promising to protect the heart against myocardial infarction (14).

As mentioned above, it is reasonable to think that tissue compression could regulate cardioprotective effect of RIC by its immunomodulatory function. To test this hypothesis, we designed a murine model showing remote conditioning using rhythmic stimulation of limbs protect myocardial infarction by the downregulation of inflammation *via* adenosine-mediated activation of A2 adenosine receptors and consequent activation of cAMP/PKA signaling pathway inhibiting NF-κB-mediated expression of inflammatory cytokines, which in favor of developing an easy implement and efficient cardiac conditioning therapy with no or fewer adverse events.

## Materials and Methods

### Experimental Animals

Adult male Sprague-Dawley (SD) rats weighing 260 ± 20 g were obtained from Charles River Laboratories (SCXK 2016-0006) and housed in a standard environment, with the temperature of 25 ± 2°C, relative humidity of 50 ± 5%, and light–dark cycle for 12 h. The study protocol was in accordance with the Guide for the Care and Use Committee at the Nanjing University of Chinese Medicine.

### Establishment of AMI in Rats

Briefly, rats were anesthetized with 5% isoflurane and oxygen with a flow rate of 0.4 L/min until the loss of righting reflex. They were then placed supine on a temperature-controlled experimental board set at 37 ± 3°C and maintained by 2% isoflurane in 100% oxygen with a flow of 0.4 L/min by means of intubation connected to a small animal ventilator (R407, RWD, China) set at a respiratory rate of 60–70 breaths per minute. After disinfecting the surgical area, a longitudinal incision was made to expose the heart. Left anterior descending (LAD) coronary artery was ligated between the pulmonary artery and the left atrial appendage with 6.0 silk suture to induce ischemia. Decreased ventricular wall motion, pale left ventricular wall, and elevated ST-segment confirmed a successful establishment of AMI model in rats.

### Remote Conditioning by Rhythmic Compression on Forelimbs of Rats

**Figure 1:**
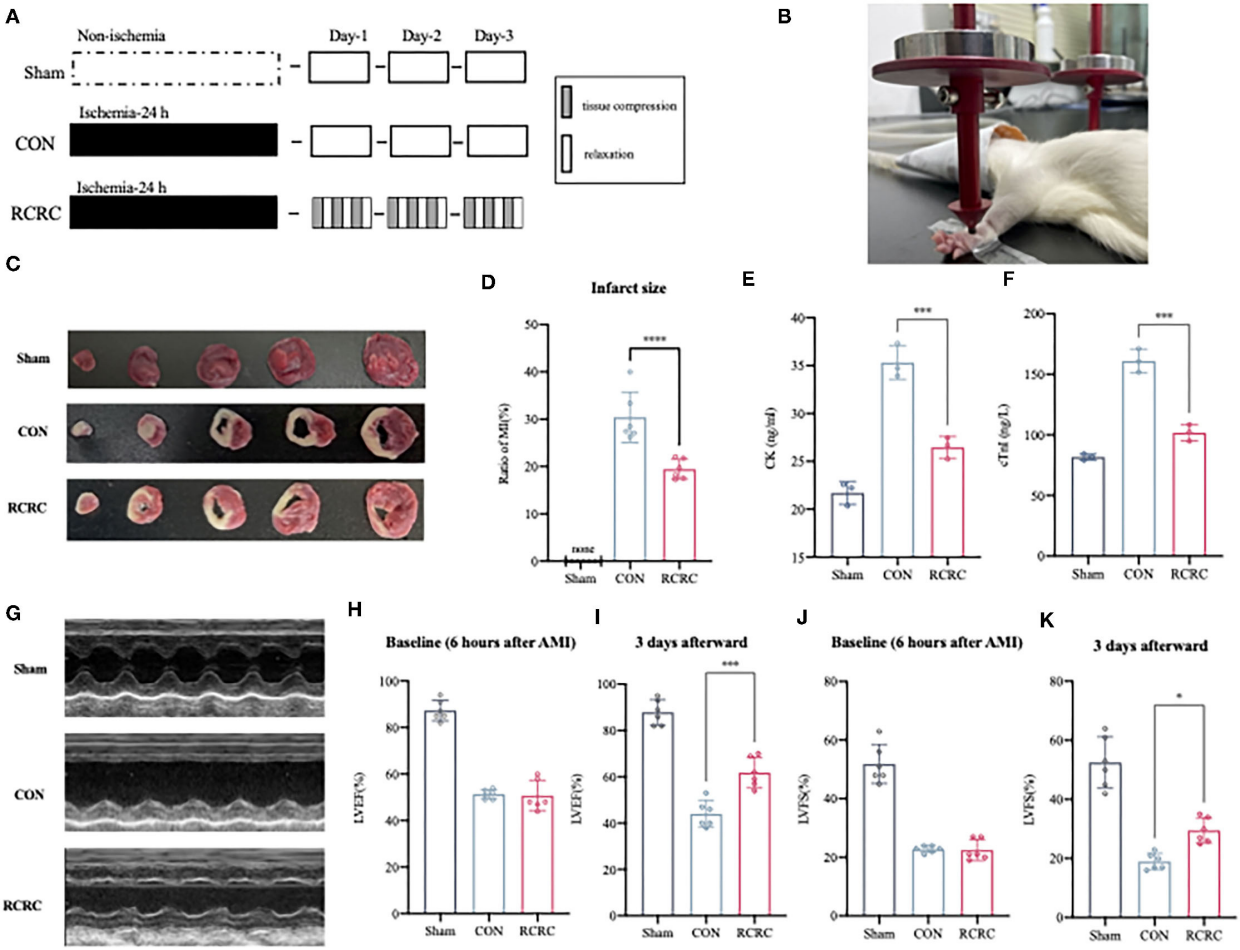
Remote conditioning by rhythmic compression decreases infarct size and ameliorates ventricular dysfunction in a rat model of AMI. **(A)** Schematic showing the overall animal study design used to test the cardioprotection of remote conditioning in a rat model of AMI. **(B)** Presentation of remote conditioning on forelimbs of rats. **(C,D)** TTC staining representing infarct area on multiples slices of an infarct rat heart and quantitative analyses of infarct size after 3 days of remote conditioning, *N* = 6. **(E,F)** Serum concentration of CK and cTnI of each group were evaluated after 3 days of remote conditioning, *N* = 3. **(G–K)** Representative M-mode echocardiographic images showing LV wall motion of the hearts and LVEF, LVFS measured by echocardiography at 6 h (baseline) and 3 days (endpoint) after MI, *N* = 6. *p < 0.05, ***p < 0.001, ****p < 0.0001 vs. CON.

Forelimb-immobilized rats were anesthetized and laid at a supine position with their shoulder joints extended, so that their facies palmaris faced upward. Remote conditioning was applied by a pair of vertically placed weight units combined with 3-mm-diameter cylindrical rubbers. Remote conditioning magnitudes depended on the quality of the weight unit, totaling 150 g. The target surface was located on the anterior forelimb, 3 mm above to the wrist joint, between the ulnar and radial sutures. The rat forepaws remained ruddy after rhythmic compression. Remote conditioning was repeated using 5 min of tissue compression and 5 min of relaxation for 3 cycles daily for 3 days (Figures 1A,B).

### Experimental Groups

All rats were randomly divided into the following groups: *Sham group—*Rats were subjected to identical surgical procedures, except for LAD ligation. *Model control group (CON)*—Rats were subjected to identical surgical procedures, which include LAD ligation. *Remote conditioning by rhythmic compression (RCRC) group*—Rats were subjected to LAD ligation plus 3 days of RCRC. *RCRC* + *SCH group*—Identical to RCRC group + administration of selective A2a antagonist SCH 58261(1 mg/kg/d, i.p.). *RCRC* + *MRS group*—Identical to RCRC group + administration of selective A2b antagonist MRS 1754 (1 mg/kg/d, i.p.). BAY + CGS group—Identical to CON group + selective A2b agonist BAY 60-6583 (1 mg/kg/d, i.v.) + selective A2a agonist CGS 21680 (0.5 mg/kg/d, i.v.). The dose of A2 receptor antagonists and agonist used here was based on the previous *in vivo* study (15–17).

### Echocardiography in Rats

On the day of LAD ligation and after 3 days of remote conditioning, rats were maintained anesthesia with 2% isoflurane and fixed in a spine position. M-mode echocardiograph was performed using a small animal ultrasound (Esaote, Italy) on the long axis of the left ventricle. Left ventricular end-diastolic and end-systolic diameters (LVEDds and LVESds), left ventricular ejection fraction (LVEF), and left ventricular fractional shortening (LVFS) were recorded.

### Measurement of Infarct Size

After 3 days of remote conditioning, triphenyltetrazolium chloride (Sigma-Aldrich) was stained to the heart, and the infarct size was measured. Briefly, after washing out the remaining blood, the heat below the ligature was cut into 5 pieces and stained with 1% triphenyltetrazolium chloride at 37°C for 15 min. The living aera is red, and the infarct area is stainless. The infarct area of the heart was calculated using digital planimetry software (ImageJ 2.1).

### Enzyme-Linked Immunosorbent Assay

The serum levels of creatine kinase (CK), cardiac troponin I (cTnI), and cardiac levels of interleukin 1 beta (IL-1β), TNF-α, adenosine, and cyclic adenosine monophosphate (cAMP) were determined using enzyme-linked immunosorbent assay (ELISA) kit (Jin Yibai Biological Technology Co. Ltd. China), according to the manufacturer's protocol.

### Western Blot

Radioimmunoprecipitation assay (RIPA) protein lysate (Beyotime, China) was used to extract the total protein in myocardial tissues. BCA protein assay (Thermo Fisher, USA) was performed using supernatants to determine the protein concentration. Protein samples were electrophoresed on polyacrylamide gels and transferred onto polyvinylidene difluoride (PVDF) membranes. After blocking with 5% nonfat milk for 2 h, the membranes were incubated with primary antibodies (Affinity Biosciences, USA) overnight at 4°C. On the next day, the membranes were incubated with secondary antibodies (Proteintech Group, USA) for 2 h. β-Actin was used as the loading control.

### Quantitative Real-Time Polymerase Chain Reaction

Total RNAs in myocardial tissues were extracted using TRIzol (Thermo scientific, USA). After reverse transcription into cDNA, quantitative real-time polymerase chain reaction (qRT-PCR) was performed to amplify the target genes. The mRNA expressions of A2a and A2b receptors were determined through Lightcycle 96 real-time-PCR system (Roche, Germany) with GAPDH as an internal control. Each sample was analyzed 3 times and the relative expressions of genes were determined using the 2 ^−ΔΔ*CT*^ method. The nucleotide sequences of primers used are shown in Supplementary Table 1.

### Statistical Analysis

Statistical analysis was performed using SPSS 26.0 software. Quantitative data were represented as mean ± standard deviation ($\bar{X}$ ± S). Student's *t*-test and/or one-way analysis of variance with multiple comparison posttest (Bonferroni) was used to compare the means between experimental groups as indicated. If the data were not normally distributed, the Kruskal–Wallis H method was used for group comparisons. A value of *p* ≤ 0.05 was considered statistically significant.

## Results

### Remote Conditioning by Rhythmic Compression Decreases Infract Size and Improves Cardiac Function in AMI Rats

To determine whether RCRC decreases infract size and improves cardiac function, rats were exposed to forelimb RCRC or CON. At 24 h post-ischemia, rats were subjected to 30-min remote conditioning (Figures 1A,B). As shown in Figures 1C,D, the infarct size was smaller in the RCRC group (19.44 ± 2.104%) than in the CON group (30.42 ± 5.331%). CK and cTnI are the important markers of AMI, so we detected serum CK and cTnI activity. Serum CK and cTnI activity was significantly increased at 3 days post-AMI that was decreased in RCRC group compared with the CON (Figures 1E,F). Therefore, remote conditioning displayed cardioprotective effect against ischemic injury. Additionally, we evaluated cardiac function by echocardiography as shown in Figures 1G–K. The baseline LVEFs and LVFSs were both similar for both CON and RCRC groups, which indicated a similar degree of initial injury (Figures 1H,J). The LVEF and LVFS were significantly declined to 44.00 ± 5.692 and 19.00 ± 2.757%, respectively, in the CON group, while they were increased to 61.83 ± 6.494 and 29.50 ± 4.231%, respectively, in the RCRC group (Figures 1I,K). The results strongly indicated that remote conditioning attenuates the deterioration of left ventricular function in AMI.

### Remote Conditioning by Rhythmic Compression Abates the Inflammatory Response in the Hearts of AMI Rats

**Figure 2:**
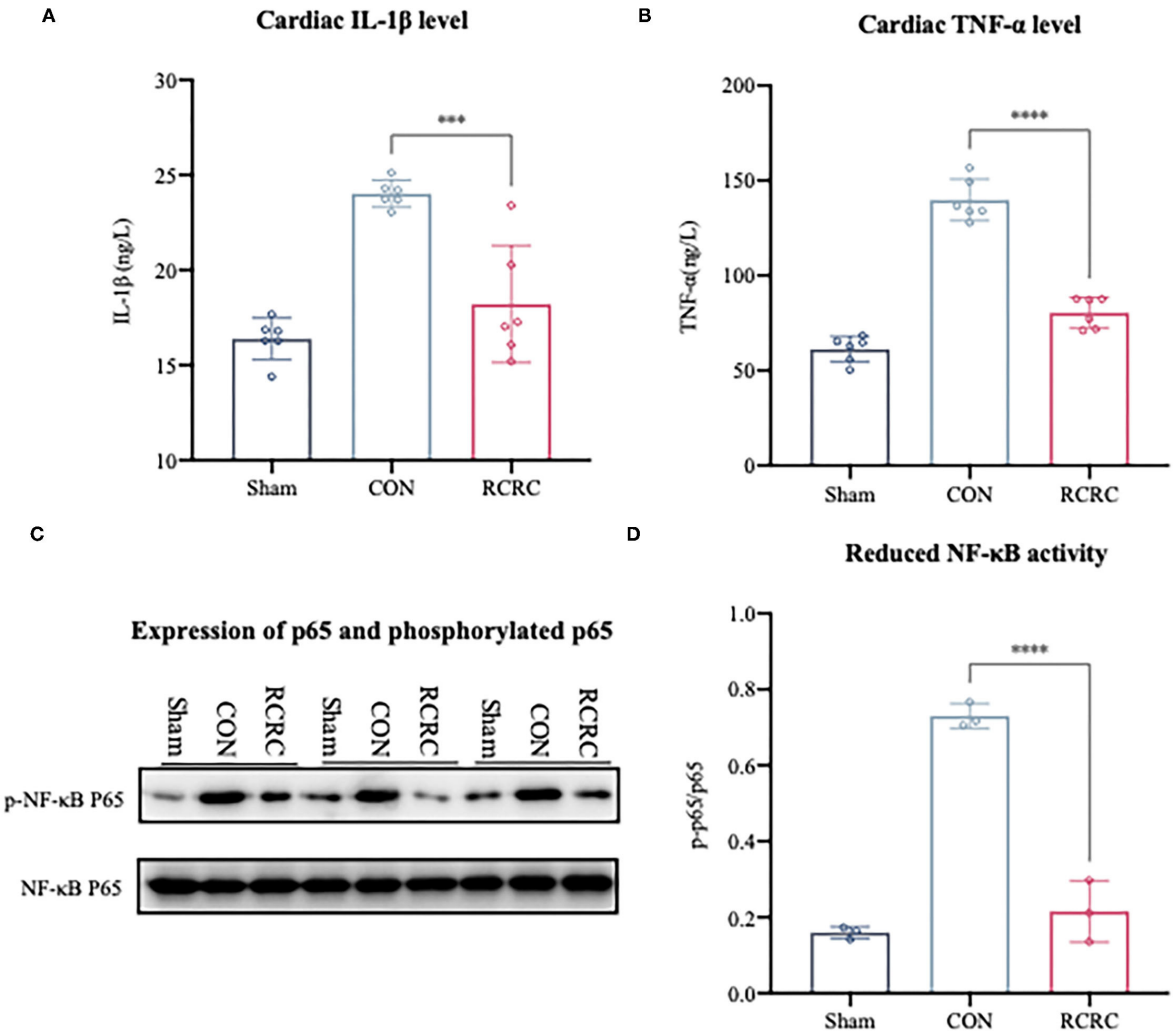
Remote conditioning by rhythmic compression abates the inflammatory response in the hearts of AMI rats. **(A,B)** The concentrations of IL-1β and TNF-α in the heart were determined by ELISA, *N* = 6. **(C,D)** The levels of phosphorylated p65 (p-p65) and total p65 in heart were detected by western blot. The corresponding quantification data of p-p65 to p65 ratio are shown in right panel. All values represent as means ± SD. *N* = 3. ***p < 0.001, ****p < 0.0001 vs. CON.

The growing evidence suggests that accentuation, prolongation, or expansion of the postinfarction inflammatory response result in worse remodeling and dysfunction after myocardial infarction (18). NF-κB is well known to be a key regulator of several kinds of inflammatory response, including postinfarction inflammation (19). Based on the observation that remote conditioning could protect myocardium against AMI, investigation of the NF-κB may help us to further explain the underlying mechanisms. Western blot analysis indicated that remote conditioning could significantly reduce NF-κB p65 phosphorylation induced by AMI (Figures 2C,D). A reduction in NF-κB activation suppresses the expression of various genes, which include TNF-α and IL-1β (20). In this study, we also found remote conditioning markedly dampened the cardiac expression of TNF-α and IL-1β, which were decreased by 42.4 and 21.57% at the day 3 (Figures 2A,B), respectively. These results confirmed that remote conditioning inhibits the postinfarction inflammatory response by inhibiting NF-κB-mediated expression of inflammatory cytokines.

### Remote Conditioning by Rhythmic Compression Induces Serum and Cardiac Adenosine Levels

**Figure 3:**
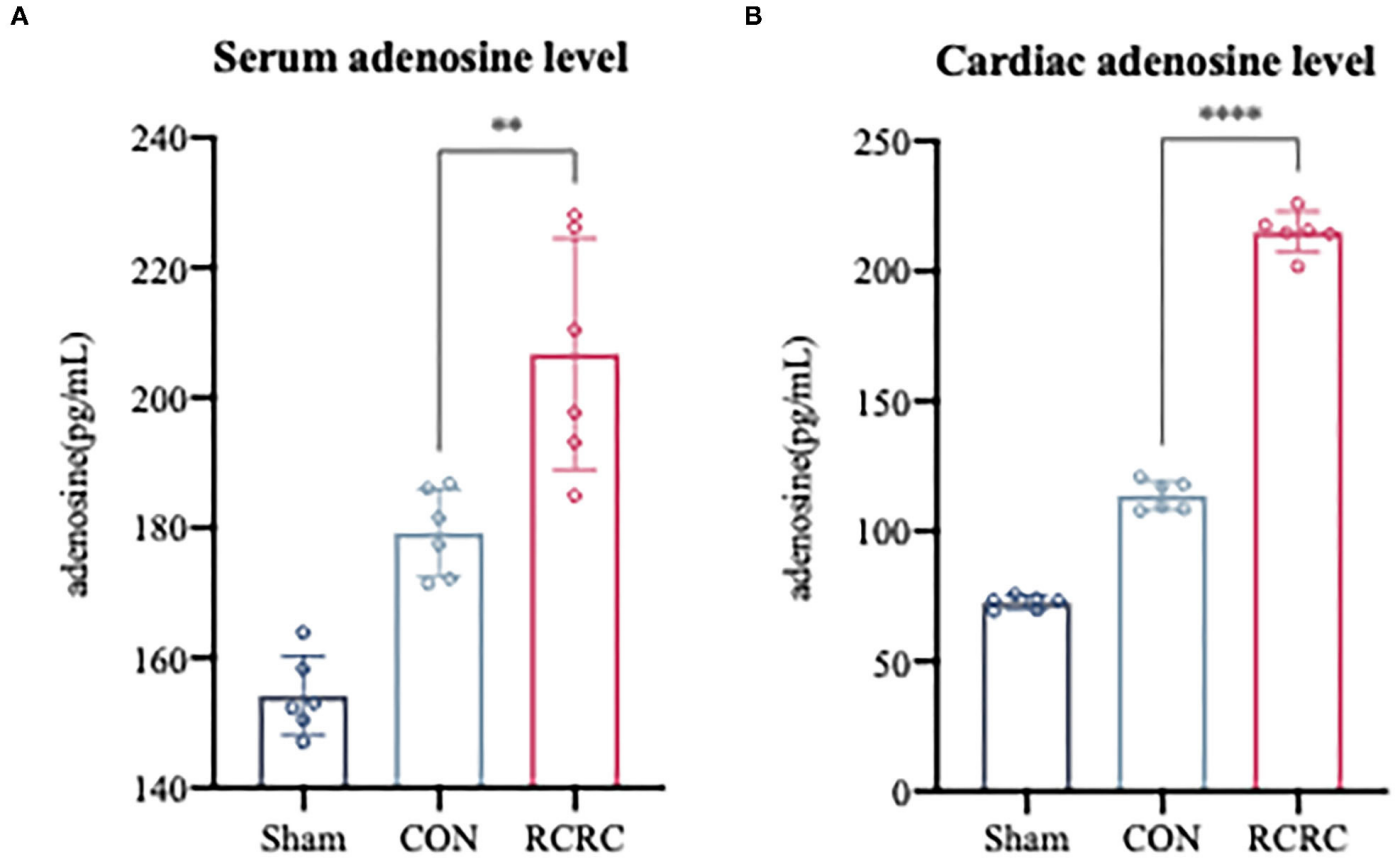
Remote conditioning by rhythmic compression increases serum and myocardial adenosine concentrations in a rat model of AMI. **(A)** Serum adenosine concentration after 3 days of remote conditioning, *N* = 6. **(B)** Adenosine concentration in infarct zone after 3 days of remote conditioning. All values represent as means ± SD. *N* = 6. **p < 0.01, ****p < 0.0001 vs. CON.

In response to all remote stimuli, there is humoral signal transfer to the target organ heart. Having established the phenomenon that remote conditioning inhibited the postinfarction inflammatory response and protected heart against AMI, we next asked whether remote conditioning induces the release of substances into the bloodstream that reach the heart to exert cardioprotective effect. Since adenosine has the potential to contribute to humoral transfer of remote conditioning and promotes tissue repair and regeneration with suppression of inflammation (21, 22), rats were exposed to sham, CON, and RCRC to determine whether remote conditioning regulates adenosine levels in the blood and heart. Serum and heart adenosine levels were measured after 3 days of remote conditioning (Figure 3). Remote conditioning applied by 5 min of tissue compression of 150 g weight unit every 10 min for a total of 30 min increased the serum adenosine levels (Figure 3A), while in the heart, remote conditioning also increased adenosine levels (Figure 3B), which suggested that upregulation of serum and cardiac adenosine levels may produce a strong cardioprotection.

### Remote Conditioning by Rhythmic Compression Is Associated With Induction of Cardiac A2 Receptors Expression and Function

**Figure 4:**
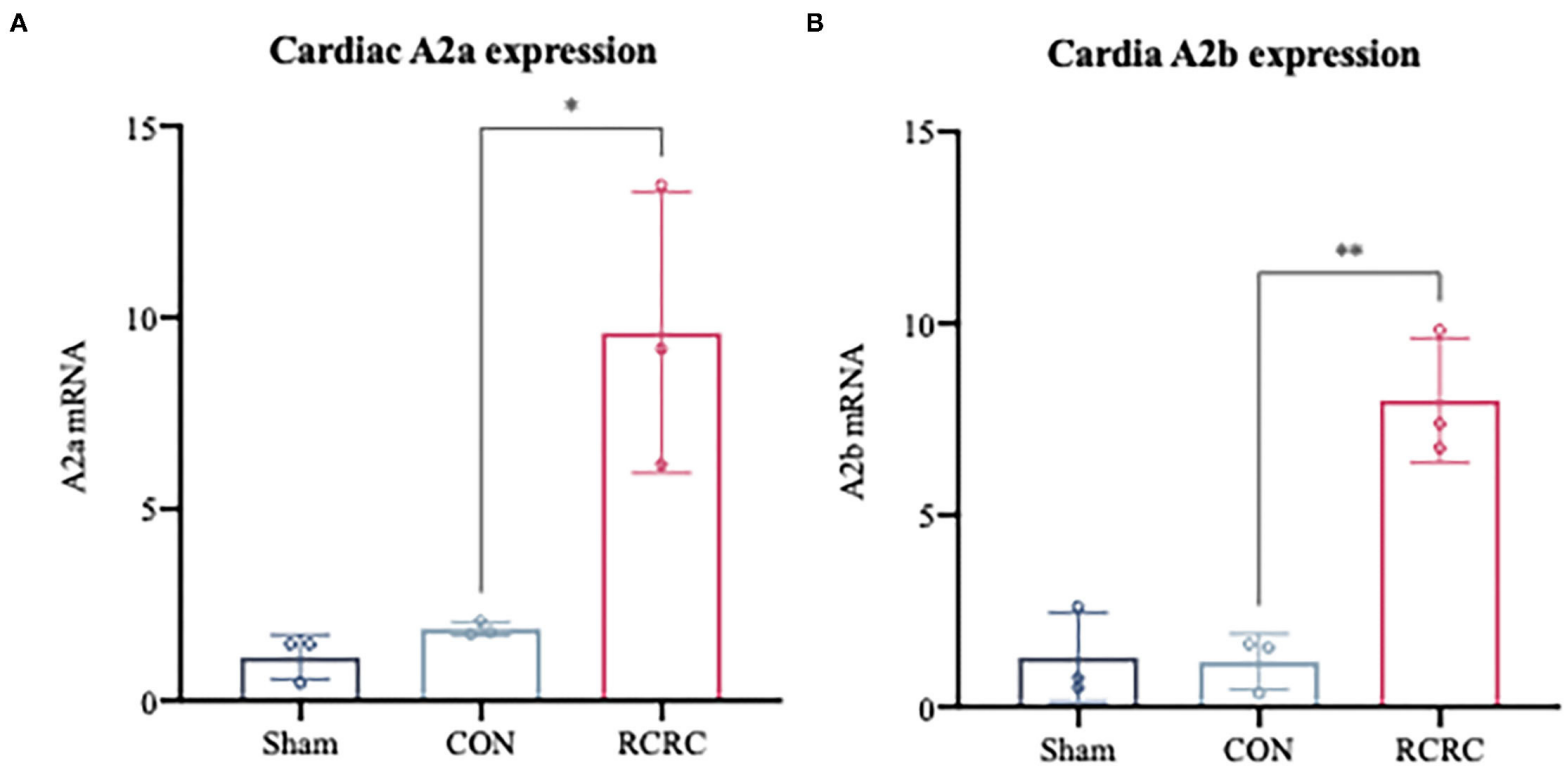
A2 adenosine receptors mRNA expression in infarct myocardium after treatment with 3 days of remote conditioning. **(A)** Relative A2a AR mRNA levels were determined by RT-PCR after treatment with 3 days of remote conditioning, *N* = 3. **(B)** Relative A2b AR mRNA levels were determined by RT-PCR after treatment with 3 days of remote conditioning. All values represent as means ± SD. *N* = 3. *p < 0.05, **p < 0.01 vs. CON.

Considering the observation that adenosine-induced cardioprotection may be possibly related to decrease in inflammation (23), we first examined the expression of A2 adenosine receptors (AR) in the infarct myocardium after 3 days of remote conditioning, which were shown to decrease the levels of inflammatory mediators in the previous studies (24, 25). As shown in Figure 4, the message for A2a and A2b receptors was significantly increased in hearts of AMI rats treated with remote conditioning, which suggested the induction of A2 receptors in the hearts during remote conditioning.

### Effect of Remote Conditioning by Rhythmic Compression on the cAMP/PKA Signaling Pathway in AMI Rats

**Figure 5:**
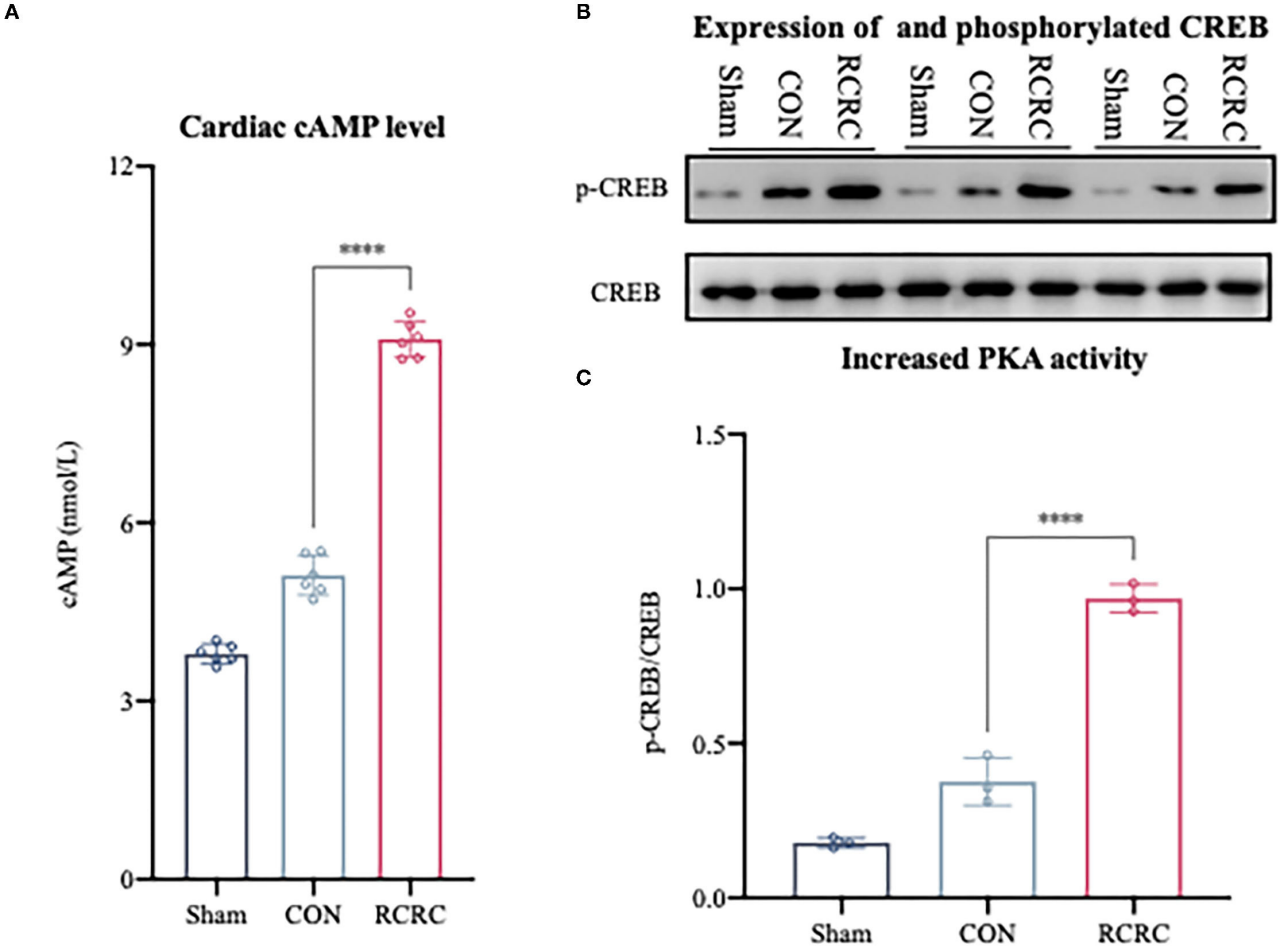
Remote conditioning by rhythmic compression reduced cAMP content and PKA activity in heart of AMI rats. **(A)** Levels of cAMP in the heart of AMI rat were determined by ELISA, *N* = 6. **(B,C)** Levels of phosphorylated CREB (p-CREB) and total CREB in heart were detected by western blot. The corresponding quantification data of p-CREB to CREB ratio are shown in right panel. All values represent as means ± SD. *N* = 6. ****p < 0.0001 vs. CON.

A2a and A2b ARs are linked to Gαs proteins, which upregulate cAMP expression (22). As we know, cAMP elevation leads to the activation of serine kinase PKA, which phosphorylates the transcription factor CREB on S133 (26). CREB phosphorylation in the infarct zone of hearts was examined after 3 days of remote conditioning to determine the PKA activity. The phosphorylation signal was significantly increased in RCRC group (Figures 5B,C), and this increment was associated with a rise in cAMP production (Figure 5A), which suggested that remote conditioning is linked with cAMP/PKA signaling pathway.

### The A2 Receptors Are Responsible for the Cardioprotective Effects of Remote Conditioning by Rhythmic Compression

**Figure 6:**
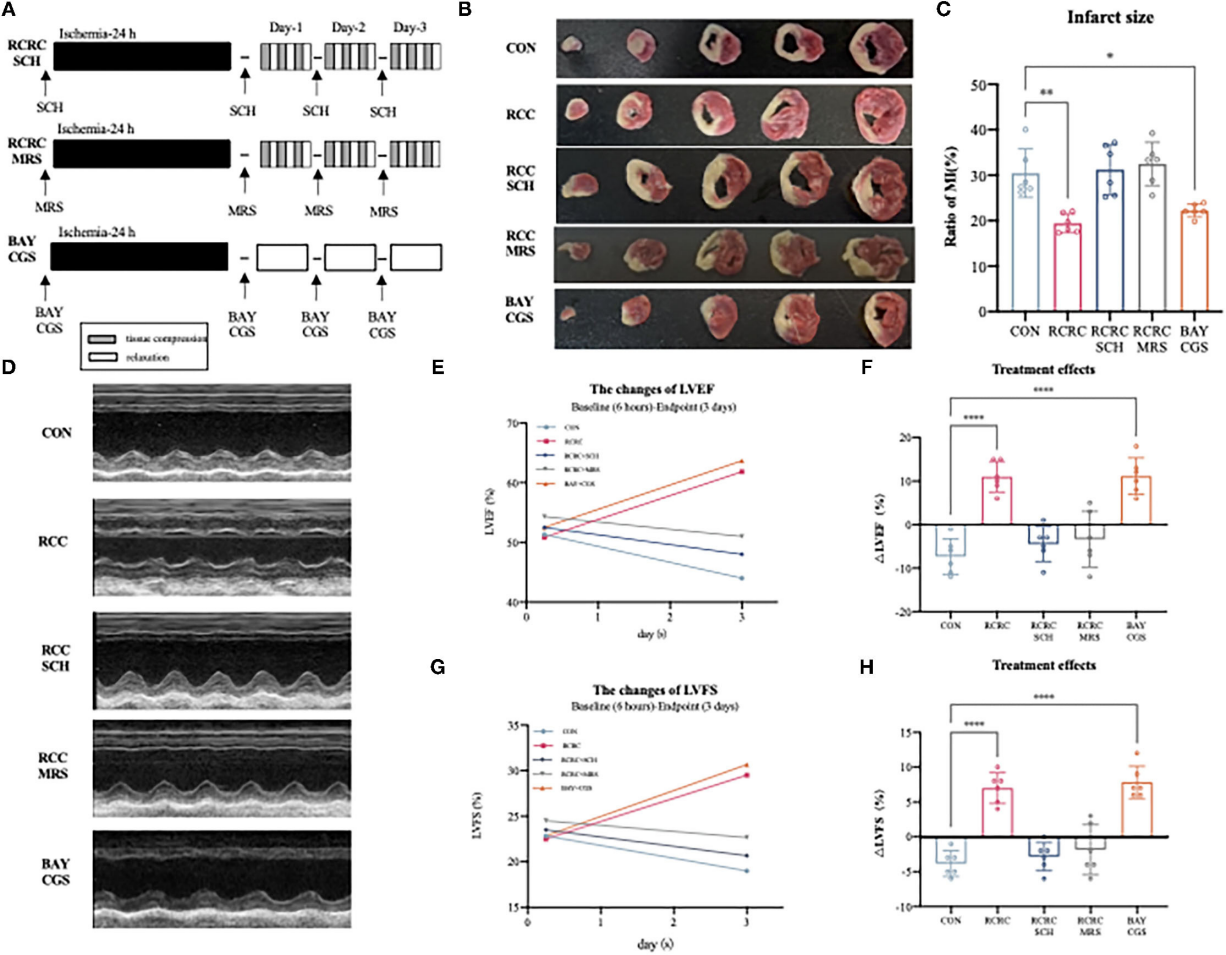
Cardioprotection of remote conditioning by rhythmic compression is lost in SCH or MRS treated rats and mimicked by BAY and CGS treatment. **(A)** Schematic diagram of the experimental design. RCRC group were treated with the selective A2a antagonist SCH 58261, the selective A2b antagonist MRS 1706; CON group treated with the selective A2a agonist CGS21680 and the selective agonist A2b agonist BAY 60-6583. **(B,C)** TTC staining representing infarct area on multiples slices of an infarct rat heart and quantitative analyses of infarct size after 3 days of remote conditioning in combination with A2a and A2b receptor blockage and activation, *N* = 6. **(D–H)** Representative M-mode echocardiographic images showing LV wall motion of the hearts, the treatment effect was calculated as the changes of LVEFs and LVFSs from baseline to endpoint in combination with A2a and A2b receptor blockage and activation. All values represent as means ± SD. *N* = 6. *p < 0.05, **p < 0.01, ****p < 0.0001 vs. CON.

To determine whether A2 receptors are critical for the cardioprotective effect of RCC, we then evaluated the impact of A2a and A2b receptors' blockage and activation on the cardioprotection of remote conditioning. A schematic diagram of the protocol is shown in Figure 6A. Combination treatment with remote conditioning and the selective A2a antagonist SCH 58261 or the selective A2b antagonist MRS 1754 reversed the anti-infarct effect compared with that in AMI rats treated with remote conditioning alone (Figures 6B,C), which indicated that both A2a and A2b receptors are involved in the action of remote conditioning. Based on the observation, we then treated AMI rats by combining the selective A2a agonist CGS 21680 and the selective A2b agonist BAY 60-6580, which reduced infarct size to 21.88 ± 1.288%, which was similar to remote conditioning (19.44 ± 2.104%), which implies that simultaneous activation of A2a and A2b receptors may produce a robust cardioprotection. The baseline (6 h after AMI) and endpoint (3 days afterward) LVEFs and LVFSs were measured as the indicators of cardiac functions in all groups. The baseline LVEFs and LVFSs were comparable for all the groups, which suggested a similar degree of initial ischemia injury (Supplementary Figure 1). Likewise, combined application of remote conditioning and SCH or MRS exhibited smaller LVEFs and LVFSs than those from the only remote conditioning group at the 3rd day (Figures 6D,E,G). However, CGS combined with BAY show a rise LVEFs and LVFSs, an effect that was equipotent with remote conditioning (Figures 6D,E,G). We also calculated treatment effect, that is, the changes of LVEFS and LVFSs from the baseline to the endpoint. While the RCRC + SCH group and RCRC + MRS group displayed a functional decline, CGS + BAY group preserved cardiac function (Figures 6D,F,H). Thus, these results suggest that A2a and A2b receptors are indispensable parts for cardioprotection of remote conditioning.

### Activation/Inhibition of A2 Receptors Affects Remote Conditioning-Regulated Release of Pro-inflammatory Factor Following Myocardial Ischemia Injury

**Figure 7:**
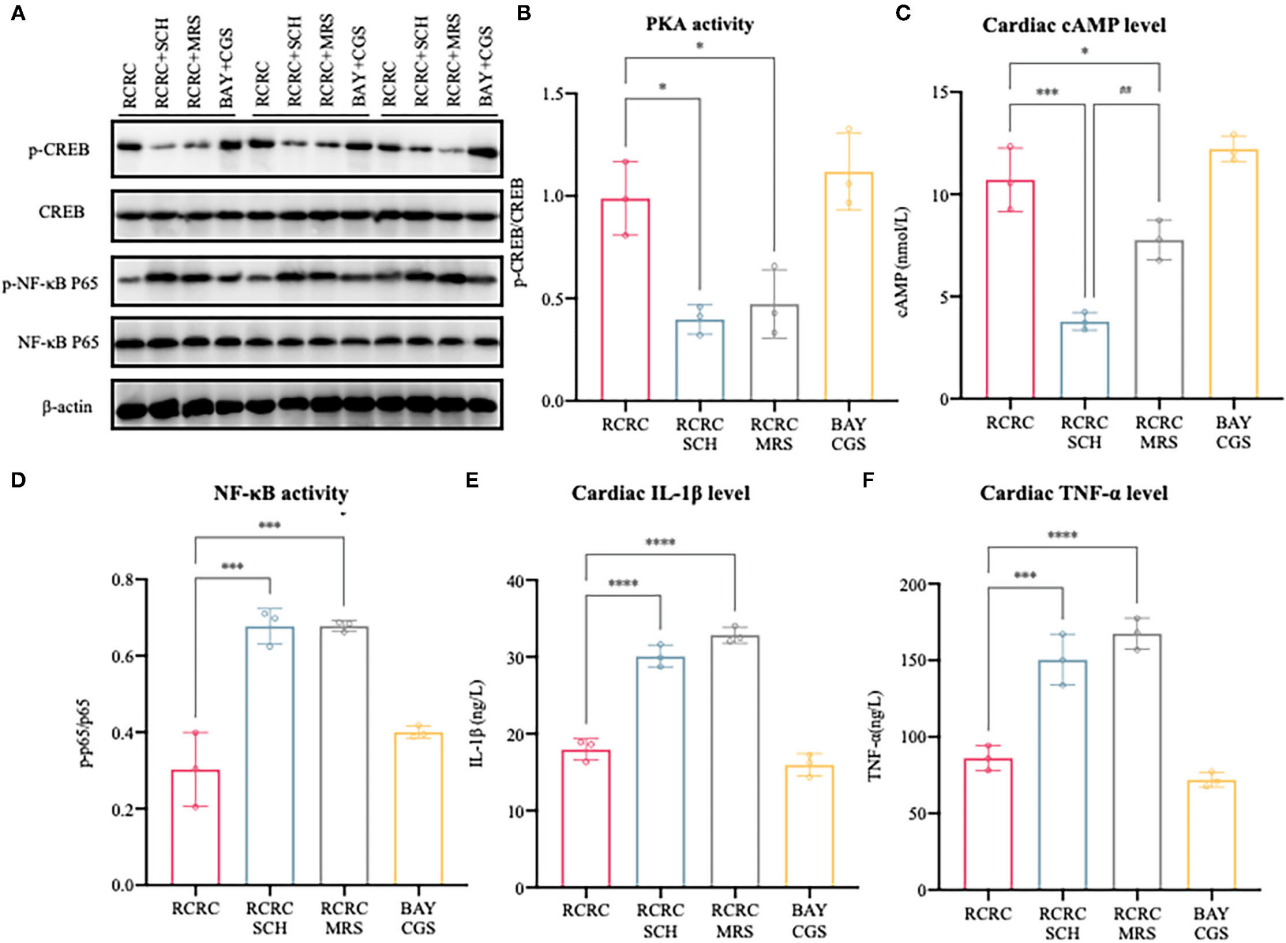
Activation/inhibition of A2 receptors affects remote conditioning-regulated release of pro-inflammatory factor following myocardial ischemia injury. **(A)** Western blot was performed to determine p-CREB, CREB, p-NF-κB p65, and NF-κB p65. **(B)** Quantitative analysis of p-CREB and CREB expression and calculate the ratio of p-CREB/CREB, *N* = 3. **(C)** Levels of cAMP in the heart of AMI rat were determined by ELISA, *N* = 3. **(D)** Quantitative analysis of p-NF-κB p65 and NF-κB p65 expression and calculate the ratio of p-p65/p65, *N* = 3. **(E,F)** The concentrations of IL-1β and TNF-α in the heart were determined by ELISA, *N* = 3. *p < 0.05, ***p < 0.001 vs. RCRC; ^##^*p* < 0.01 vs. RCRC+SCH, ****p < 0.0001.

To further determine the causal relationship between remote conditioning and A2 receptors, we also identified the downstream signal pathway following antagonization and inhibition of A2 receptor. As shown in Figures 7A–C, treatment with the selective A2a antagonist SCH 58261 or the selective A2b antagonist MRS 1754 reduced cAMP expression and PKA activity caused by remote conditioning considerably, which suggested that remote conditioning plays its role through A2 receptors. Meanwhile, the selective A2a agonist CGS 21680 and the selective A2b agonist BAY 60-6580 mimicked the cAMP expression and PKA activity of remote conditioning, lending weight to the preceding deduction. These results demonstrate the functional activation of A2 receptors by remote conditioning.

In addition, treatment with either SCH 58261 or the MRS 1754 significantly blocked the anti-inflammatory action of remote conditioning as evidenced by the decreased expression of TNF-α, IL-1β, and NF-κB p65 phosphorylation, although this was not observed with A2 agonists (Figures 7D–F). According to the above study, the A2 ARs are involved in mediating cAMP/PKA signaling pathways that inhibit NF-κB-mediated expression of inflammatory cytokines. In summary, remote conditioning attenuated MI-induced inflammation and myocardial injury *via* A2 receptors.

## Discussion

A growing body of evidence points the cardioprotective effect of RIC and related signal transduction pathways (27). However, there is currently nothing known about the cardioprotection of mechanical tissue compression, which is involved in composing RIC. In this study, we first report to our knowledge, the cardioprotective effects of RCRC on forelimbs. We demonstrated that remote conditioning reduces infarct size and attenuates left ventricular dysfunction, by decreasing postinfarction inflammatory response. Then, we showed that remote conditioning increased plasma and cardiac adenosine levels after 3 days of remote conditioning. Meanwhile, remote conditioning also prompted the expression of cardiac A2 receptors and its subsequent cAMP/PKA/CREB signaling pathway. With the background, we shed light on the importance of A2 receptors on the cardioprotective effect of remote conditioning. The results showed that the inhibition of either A2a or A2b receptor alone abolished the cardioprotection of remote conditioning and simultaneous stimulation of both A2 receptor subtypes contributed to a robust cardioprotective effect against AMI, which collectively demonstrated that A2a and A2b receptors work in concert to confer cardioprotection of remote conditioning. Thus, our study has demonstrated for the first time that remote conditioning confers cardioprotection by upregulating levels of adenosine, as a humoral factor and activating cAMP/PKA/CREB signaling pathway through A2 receptors in the heart.

Remote ischemic conditioning by brief episodes of ischemia with reperfusion on an extremity displayed a robust reduction of infarct size and improvement of the prognosis in patients with AMI (3, 28). However, accumulating evidence suggests that transient ischemia is not a requisite trigger for remote cardioprotection (29). RIC is now considered as a systemic response, which could be mimicked by various stimuli (3). For instance, trauma by transverse abdominal skin incision, partly a mechanical stimulus, contributes to a myocardial infarct-limiting effect in rodents and dogs (30–32). In fact, RIC is a stimulus compound containing interruption of blood vessel, edema, and tissue compression. In this study, we separated the above factors and only focused on the tissue compression, to extend into the novel concept of remote protection in AMI. Our results presented for the first time that tissue compression, a form of mechanical stimulus involved in RIC (33), also showed cardioprotective effect against AMI.

Myocardial infarction induces an inflammatory response that removes the wound from dead cells and matrix debris, while prolonged or expanded inflammation would contribute to the adverse outcome (18). To find out its cause, inflammation to injury is evolved to protect organisms against infectious pathogens, which may be excessive for the delicate requirements of the ischemic myocardium. In other words, cardiac repair is dependent on a terrifically orchestrated inflammatory reaction. Accumulating evidence suggests that appropriate and timely containment and resolution of inflammatory response are the determinants of the quality of cardiac repair (34, 35). As previously described, mechanical stimuli, such as massage, are generally recognized to be beneficial for reducing inflammation, particularly reflecting on tempering the increase of pro-inflammatory mediators and inflammatory cell infiltration (6, 36). As an important upstream regulator of the inflammation, activated NF-κB upregulates the transcription of target genes, for example, TNF-α and IL-1β, which are linked to the progression and prognosis of AMI (37). This study showed that remote conditioning inhibited the activation of NF-κB and lowered the expression of pro-inflammatory cytokines (TNF-α, IL-1β), which may help to ameliorate the susceptibility of AMI.

It is proposed that regulation of the immune responses needs at least two “danger” signals. Aside from the first danger signal leading to the activation of immune cells, there must be a second danger signal, which could downregulate the inflammation to protect against the excessive collateral damage and the destruction of normal tissues (13). Extracellular ATP accumulation frequently induces inflammation, which reflects metabolic alterations caused by cellular stress (38). The hydrolysis of ATP to adenosine, which represents such earliest “second danger signals,” has immunosuppressive action on immune cells and hence protects tissues from excessive inflammation (13, 38). In fact, adenosine is a purine nucleoside that is broadly distributed in a variety of tissues and body fluids and plays a vital role in different physiological and pathological conditions (23, 39). Here, we demonstrated that remote conditioning induced adenosine liberate into the blood and increased the concentration of it in the heart, for defining the signal transduction of remote conditioning from the trigger limbs to heart.

Extracellular adenosine may, in a way, fulfill its supposed role as a “retaliatory” metabolite in protecting the heart and other tissues from, for example, ischemia damage as the first signal of danger that reports excessive immune damage to normal tissues by hyperactive immune cells (40). It has been reported that adenosine may be associated with ischemic conditioning and its release could confer cardioprotective effect (41). In mice, adenosine levels in arterial plasma were increased after persistent brain ischemia by bilateral ligation of the internal carotid arteries, and this increment was subsequently linked to infarct-limiting effect in isolated perfused mice hearts (42). It is also documented that exogenous of adenosine mimicked the cardioprotective effects of ischemic and remote preconditioning (43). Based on the previous findings and this study, we proposed that adenosine is the key humoral factor of remote cardioprotection, which could act as a “reporter” of metabolic changes.

Adenosine produces its pharmacological effects mainly through its interaction with AR: A1, A2a, A2b, A3, which are all members of the G-protein-coupled receptors family (44). A1 and A3 ARs are coupled to Gi/o, through which they reduce cAMP levels, while Gs protein coupled A2a and A2b ARs stimulate adenyl cyclase (AC) and cause accumulation of intracellular cAMP, which possesses immunosuppressive effects (13). In this study, we demonstrated that remote conditioning increases cardiac cAMP production, which suggested the activation of A2 receptors from a functional perspective. Follow this approach, we detected the expression of A2 receptors in the heart and witnessed a rise in the levels of A2 receptors, which could act as “sensors” of metabolic changes.

From this point, it is necessary to determine whether A2 receptors are indispensable parts of cardioprotection during remote conditioning. Interestingly, the activation of either A2a or A2b receptor alone was unable to decrease infarct size in the CD73^−/−^ mice, which could not produce large amounts of adenosine (45). In this study, we showed that coadministration of remote conditioning and the selective A2a antagonist SCH or the selective A2b antagonist MRS led to the loss of infarct-limiting effect and cardiac functions. Meanwhile, the combination of the selective A2a agonist CGS and the selective A2b agonist BAY reduced infarct size and improved cardiac functions. These results clearly indicated that simultaneous stimulation of A2a and A2b ARs is critically important for the cardioprotection of remote conditioning, as revealed in the preliminary evidence (14, 46). But why both A2a and A2b receptors are required to produce cardioprotective effect? The most obvious reason is that both receptors are coupled to Gs protein and share the mutual downstream pathway. One possible answer may be that A2a receptor has high affinity and A2b receptor has low affinity of adenosine, which enables the graded escalation of inhibitory signals (13). More specifically, it is the accumulation of adenosine and stepwise recruitment of A2a and then of the A2b that guarantee the option of partially retarding immune cells to continue pathogen destruction but with less tissue damage (13). In this study, although cAMP expression was reduced by treatment with both A2 antagonists, A2a antagonist reduced it more than A2b antagonist (Figure 7C). The part of the reasons might be that A2a receptor signaling can contribute to A2b expression (47). In addition, A2a receptor may play a part in both transduction and reinforcement of the cardioprotective signal from A2b receptors (14). Our subsequent study should investigate these findings.

As mentioned above, remote conditioning inhibited the activation of NF-κB and lowered the levels of TNF-α and IL-1β, which is in line with the anti-inflammatory properties of A2 receptors. More generally, A2a receptor could produce a protective function through transforming macrophage form inflammatory to angiogenic phenotypes (48). It has been also shown that A2b cardioprotective effect may be bound up with the adjustment of TNF-α and neutrophil function (49). In this study, we demonstrated that remote conditioning induced the PKA activity, which is consistent with a rise in cAMP production. In fact, the activity of NF-κB is modulated by cAMP/PKA/NF-κB axis, in which PKA activation could inhibit the activation of NF-κB (50). Therefore, cAMP-dependent PKA phosphorylates and activates CREB, thereby inhibiting the activation of NF-κB and reducing the production of TNF-α and IL-1β, which may be the mechanism beneath the cardioprotective effect of remote conditioning *via* A2a receptors.

In conclusion, we identified remote conditioning as a novel cardioprotective intervention in a murine model of AMI. This protection is induced, at least partially, by its anti-inflammatory actions in acute ischemic cardiac tissue damage. The signal transduction of remote conditioning from the trigger limb to heart involves the release of adenosine, which activated the synergistic reaction of A2a and A2b receptors, which are indispensable parts for cardioprotection of remote conditioning. Subsequently, A2 receptors activate cAMP/PKA/NF-κB axis to exert anti-inflammatory properties, which in turn contribute to cardioprotection. Our results established a new connection between remote tissue compression and cardiovascular diseases, which enhances our cognition about the role of tissue compression on RIC cardioprotection. These findings may provide a cue to developing the potential of remote conditioning as an immunomodulation procedure for cardioprotection and harnessing its effect toward a novel therapy.

## Data Availability Statement

The original contributions presented in the study are included in the article/Supplementary Materials, further inquiries can be directed to the corresponding author/s.

## Ethics Statement

The animal study was reviewed and approved by Care and Use Committee of Nanjing University of Chinese Medicine.

## Author Contributions

SX, RG, HZ, and YG designed the study. SX and RG performed experiments. XB, XX, XFX, YL, and SL performed the measurements, collected, and analyzed the data. SX wrote the manuscript. All authors approved the final version of manuscript submitted.

## Funding

This work was supported by Jiangsu Leading Talents Project of Traditional Chinese Medicine SLJ0226, Third Open Project of Nursing Advantage Subject in Nanjing University of Chinese Medicine (2019YSHL044 and 2019YSHL051), National Natural Science Foundation of China (81974583 and 81704169), and Natural Youth Fund Project of Nanjing University of Chinese Medicine (NZY81704169).

## Conflict of Interest

The authors declare that the research was conducted in the absence of any commercial or financial relationships that could be construed as a potential conflict of interest.

## Publisher's Note

All claims expressed in this article are solely those of the authors and do not necessarily represent those of their affiliated organizations, or those of the publisher, the editors and the reviewers. Any product that may be evaluated in this article, or claim that may be made by its manufacturer, is not guaranteed or endorsed by the publisher.
