# Supplementary material for: Remote Conditioning by Rhythmic Compression of Limbs Ameliorated Myocardial Infarction by Downregulation of Inflammation via A2 Adenosine Receptors
Source: Front Cardiovasc Med. 2022 Apr 8;8:723332. doi: 10.3389/fcvm.2021.723332 (PMC9040771; doi:10.3389/fcvm.2021.723332)
Supplement: Supplementary file 1 [file Data_Sheet_1.docx]

Supplementary Material

# Supplementary Table 1. Primers used in quantitative real-time polymerase chain reaction (qRT-PCR)

| Gene | Sequence (5’-3’) |
| --- | --- |
| A2a | Forward: |
|  | TTCCACTCCGGTACAATGG |
|  | Reverse: |
|  | CTGACTGCAGTTGTTCCAG |
| A2b | Forward: |
|  | GAGCTCCATCTTTAGCCTC |
|  | Reverse: |
|  | ACCAAACCTTTATACCTGAGC |

## Supplementary Figure 1

**Supplementary Figure 1. The baseline (6 hours after AMI) LVEFs and LVFSs were measured in all groups**. (A) The LVEFs at baseline were similar across all the groups. (B) The LVFSs at baseline were similar across all the groups. All values represent as means±SD. N=6 animals per group.
