# Supplementary figures and images for: Remote Conditioning by Rhythmic Compression of Limbs Ameliorated Myocardial Infarction by Downregulation of Inflammation via A2 Adenosine Receptors
Source: Front Cardiovasc Med. 2022 Apr 8;8:723332. doi: 10.3389/fcvm.2021.723332 (PMC9040771; doi:10.3389/fcvm.2021.723332)

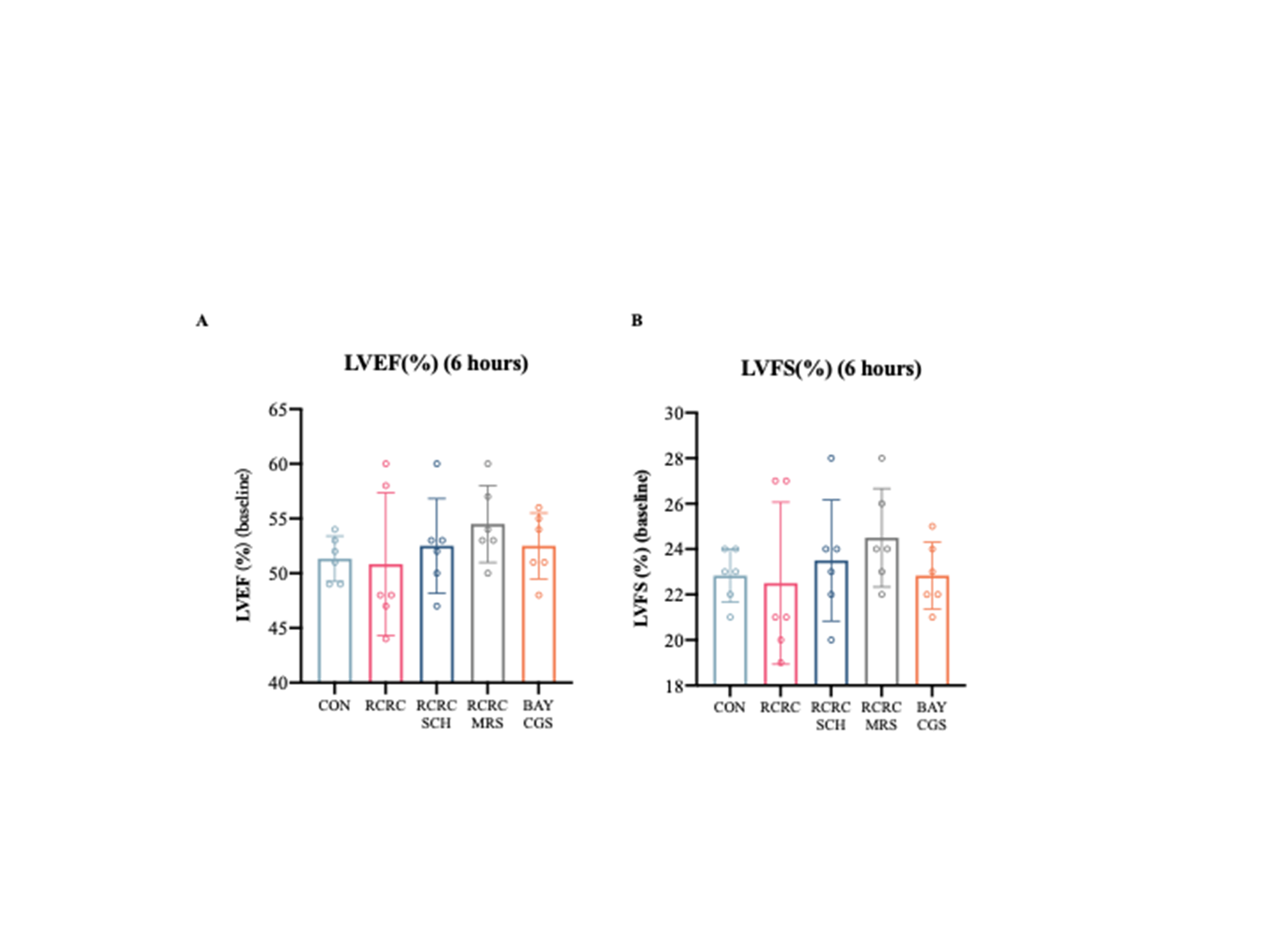

Supplement: Supplementary file 2 [file Image_1.TIFF]
